# A comparative analysis and guidance for individualized chemotherapy of stage II and III colorectal cancer patients based on pathological markers

**DOI:** 10.1038/srep37240

**Published:** 2016-11-15

**Authors:** Yang Han, Su Lu, Fudong Yu, Xisheng Liu, Huimin Sun, Jingtao Wang, Xingwu Zhu, Huijun Lu, Hao Yue, Jing Wang, Jun Lin, Chongzhi Zhou, Huamei Tang, Zhihai Peng

**Affiliations:** 1 Department of General Surgery, Shanghai General Hospital, School of Medicine, Shanghai Jiao Tong University, Shanghai 20080, China; 2 Department of Pathology, Shanghai General Hospital, School of Medicine, Shanghai Jiao Tong University, Shanghai 20080, China.

## Abstract

Adjuvant chemotherapy is considered the standard of care for patients with colorectal cancer after curative resection. Although current guidelines provide clear instructions for chemotherapy for stage II high-risk and stage III colorectal cancer, it is insufficient to individualize therapy. We analyzed the outcomes of 902 patients with colorectal cancer treated with or without chemotherapy in our hospital. We found Chinese survival benefit for chemotherapy was consistent with current guidelines. Moreover, our data added to the evidence that chemotherapy might be used for elderly patients with stage II high-risk colorectal cancer. Pathological markers could predict response to individualize therapy in a convenient, fast and inexpensive way. We compared survivals of patients with stage II high-risk and stage III colorectal cancer with chemotherapy in different pathological markers expression, and furthermore used 458 colon adenocarcinoma samples from The Cancer Genome Atlas to verify our preliminary results. We confirmed TOPIIα, EGFR and P170 may be sufficiently predictive markers to individualize chemotherapy. FOLFOX was the optimal adjuvant chemotherapy for patients with stage II high-risk and stage III colorectal cancer when TOPIIα was positive or EGFR or P170 was negative.

Colorectal cancer (CRC) is the third most commonly diagnosed cancer and leading cause of cancer death in the world^1^,^2^. China is among the countries with the highest incidence, accounting for 18.6% of newly diagnosed cases worldwide every year. With change in lifestyle, improved early screening and especially the use of fluorouracil (FU)-based adjuvant chemotherapy, colorectal cancer mortality rates have decreased worldwide^3^,^4^,^5^.

Adjuvant chemotherapy (AC) is considered the standard of care for patients with stage III CRC after curative resection^6^. Fluorouracil plus folinic acid in combination with oxaliplatin (FOLFOX) or capecitabine plus oxaliplatin (CapeOX) is an established first-line therapy for stage III colorectal cancer^7^,^8^,^9^,^10^. Single agent fluorouracil plus folinic acid (5-Fu/LV) or capecitabine is also recommended for patients who oxaliplatin is unsuitable^10^.

However, many patients do not benefit from chemotherapy. The benefit of chemotherapy for patients with stage II CRC remains under debate^11^. The advantages of chemotherapy for elder patients at the cost of increased toxicity, particularly paresthesias, bone marrow toxicity, and gastrointestinal toxicity are controversial^12^. Furthermore, CRC is a heterogeneous disease with widely varied outcomes^13^. Even patients with similar clinical and pathological features treated with the same chemotherapy may have different outcomes. Therefore, the individualized chemotherapy for patients with CRC emerges as the times of precision medicine. However, there is no simple means to individualize chemotherapy for every patient. Recently detection mutations of KRAS, NRAS or BRAF have been proven predictive of whether or not to use cetuximab or panitumumab in combination with standard chemotherapy for individual CRC patients^10^,^14^. But the NCCTG intergroup phase III trial N0147 and PETACC-8 trial showed no additional benefit to add these targeted agents to standard chemotherapy^15^,^16^,^17^,^18^. Therefore, predictive markers for benefit of adjuvant chemotherapy for CRC are lacking.

Molecular pathology is an emerging and crossover discipline within pathology that is focused on the study and diagnosis of disease through the examination of molecules^19^. Such as Her-2, EGFR, Ki67 are common and typical pathology molecules, that are routinely tested by immunohistochemistry in the department of pathology of every hospital as required to diagnose CRC. Moreover, these pathological molecular markers may not only improve more accurate diagnosis of subgroups of CRC and identify tumor characteristics, but also guide clinical therapy for CRC in a convenient, fast and inexpensive way^20^. Therefore, pathology molecular markers may help us to select optimal chemotherapy, maximizing the benefit of treatment and minimizing toxicity, potentially serving as predictive markers for individualized chemotherapy^21^.

In this study, we analyzed the outcomes of 902 patients with CRC treated with or without adjuvant chemotherapy after curative resection in our hospital to validate whether the results of Chinese patients of different stages and ages survival benefit with chemotherapy are in accordance with current National Comprehensive Cancer Network (NCCN) guidelines. Furthermore, we explored methods to guide individualized chemotherapy for stage II high-risk and stage III patients with CRC by comparing survivals based on their expressions of various pathological markers. Finally, we used 458 colon adenocarcinoma samples from The Cancer Genome Atlas (TCGA) to verify our preliminary results and confirmed that TOPIIα, EGFR and P170 could be sufficiently predictive markers for individualized chemotherapy.

## Results

### Patients characteristics

The clinical characteristics of the 902 patients are outlined in Table 1. Of the 902 patients, 633 were less than 75 years of age. There were slightly more women than that men (50.3% vs. 49.7%). The rectum was the most common CRC location (38.6%), followed by the sigmoid colon (24.3%). Patients with stage II and III CRC accounted for 72.1% of all the cases. Moreover, a total of 603 patients (66.9%) received adjuvant treatment. In a word, the composition of our cohort was consistent with that of the Chinese CRC population^22^.

To explore our cohort’s representation, we used Cox proportional hazards model to analyze independent prognostic factors (Supplementary Table 1). Univariate analysis revealed that both the OS and DFS of these samples were associated with age (P = 0.005; P = 0.006, respectively), tumor size (P = 0.028; P = 0.031, respectively), tumor location (P = 0.017; P = 0.020, respectively), pT stage (P = 0.004; P = 0.005, respectively), pN stage (P < 0.001), distant metastasis (P < 0.001), AJCC stage (P < 0.001), differentiation (P = 0.014; P = 0.015, respectively). Moreover, the multivariate analysis showed that only age (P = 0.004) and AJCC stage (P < 0.001) remained significantly independent prognostic factors, which was in accordance with other studies of large cohorts of CRC patients^23^,^24^.

Furthermore, the mean OS of the patients without chemotherapy was 62.32 months (Supplementary Fig. 1). T stage, AJCC stage and chemotherapy were mainly independent factors resulting in poor prognosis (OS < 62.32 months) for CRC patients by univariate and multivariate analysis (Supplementary Table 2).

### AC Treatment Characteristics

**Figure 1:**
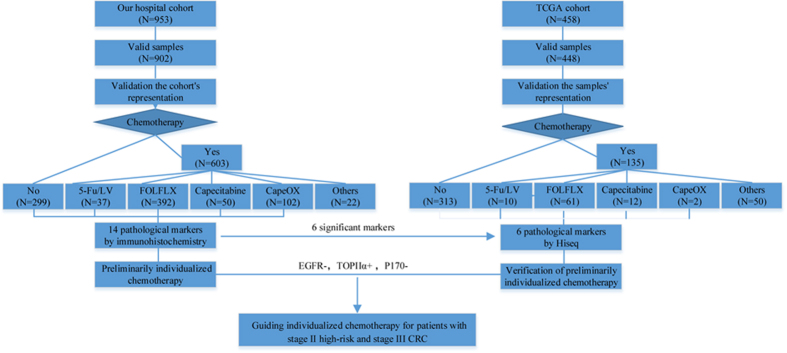
Summary description of the retrospective study regarding adjuvant chemotherapy. (**A**) The flow chart for our hospital’s cohort. (**B**) The flow chart for TCGA samples.

The detailed AC treatment characteristics of these 603 patients are presented in Fig. 1 and Supplementary Table 3. There were twelve chemotherapy regimens used for adjuvant treatment after curative resection in our cohort. Five kinds were single-agent chemotherapies and the remainders were combination therapies. However, certain chemotherapies were only used for few patients, which belonged to an individual. In our final analysis, only four chemotherapy regimens were included: 5-Fu/LV (37 patients), FOLFOX (392 patients), capecitabine (50 patients) and CapeOX (102 patients).

Moreover, the five-year OS of the patients with chemotherapy was 0.811, which was obviously higher than the ones without chemotherapy (Supplementary Fig. 1). However, there were still many patients with no benefit from chemotherapy. Those patients that did not gain survival benefit with these four chemotherapy regimens could result from advanced AJCC stage and chemotherapy resistance through univariate and multivariate analysis (Supplementary Table 4).

### AC treatment and tumor stage

**Figure 2:**
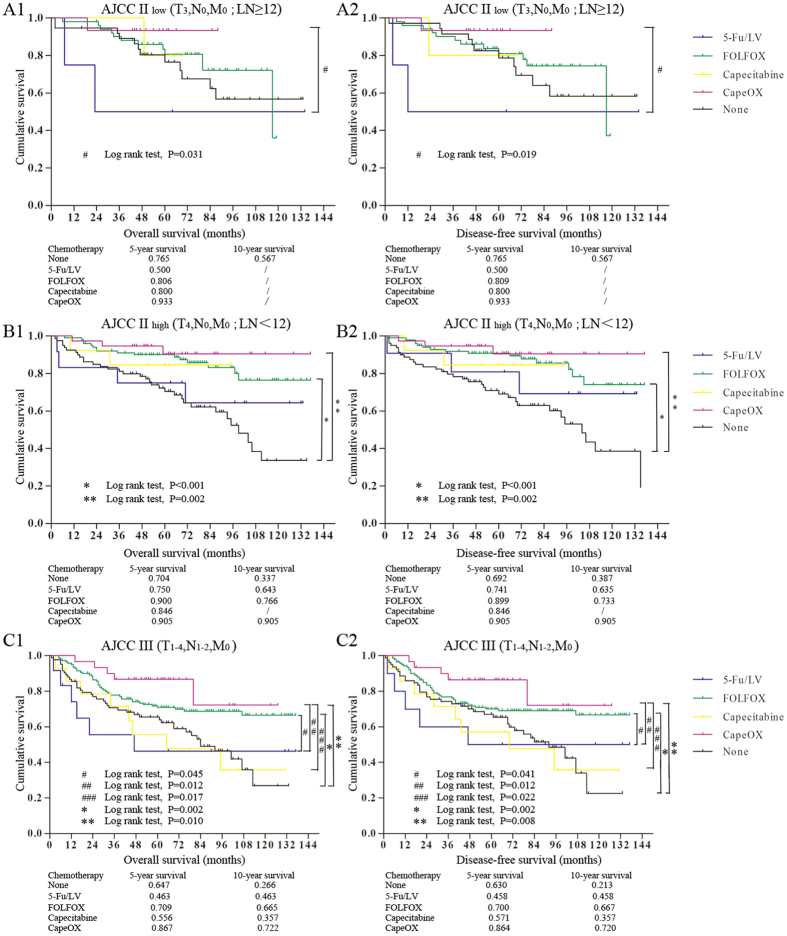
The Kaplan-Meier survival comparison of the patients with stage II and III with chemotherapy. (**A**) The overall survival and disease-free survival of stage II low-risk CRC. (**B**) The overall survival and disease-free survival of stage II high-risk CRC. (**C**) The overall survival and disease-free survival of stage III CRC.

The patients with stage III CRC treated with FOLFOX or CapeOX had substantially better survivals than those not treated with chemotherapy (70.9% vs. 86.7% vs. 64.7%) (Fig. 2C). However, the patients treated with single-agent chemotherapy, such as 5-Fu/LV or capecitabine, did not gain a survival benefit, but rather had lower 5-year OS or DFS rates, compared with those without chemotherapy (46.3% vs. 55.6% vs. 64.7%). Moreover, the patients treated with chemotherapy had an obviously superior long-term outcome, especially those with FOLFOX or CapeOX. But there was no significant difference between the FOLFOX and CapeOX groups.

The survival of patients with stage II CRC was definitely better than that of stage III patients (72.3% vs. 64.7%). The benefit of chemotherapy for patients with stage II CRC remains controversial. To explore the effect of chemotherapy in stage II CRC, we divided the stage II cases into high and low risk groups to compare their survival curves with or without chemotherapy. The definition of high-risk stage II CRC is based on the latest NCCN guidelines^10^. Chemotherapy provided a survival benefit only for stage II high-risk CRC, not for low-risk (Fig. 2A). The patients with stage II high-risk CRC with FOLFOX or CapeOX showed better outcomes compared those without chemotherapy (90% vs. 90.5% vs. 70.4%) (Fig. 2B). FOLFOX or CapeOX provided a significantly better long-term survival benefit for the patients with stage II high-risk CRC. There was still no significant difference between the FOLFOX and CapeOX groups.

Based on these results, we recommend FOLFOX or CapeOX for the patients with stage III and stage II high-risk CRC. The patients with FOLFOX or CapeOX gained a significantly better long-term survival benefit than those without chemotherapy.

### AC treatment and age

**Figure 3:**
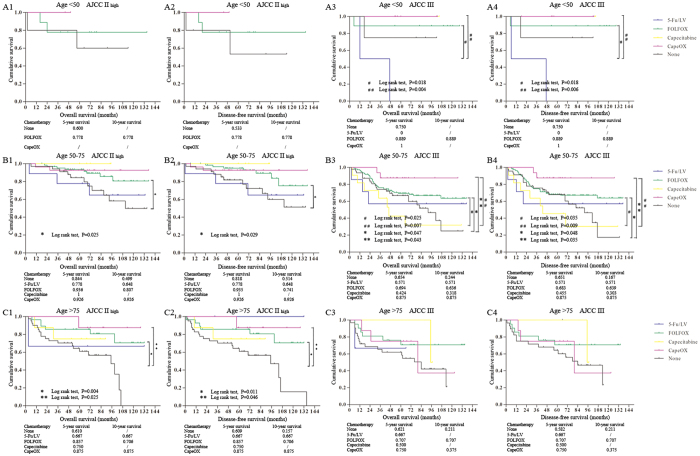
The adjusted Kaplan-Meier survival comparison of different aged patients with stage II high-risk and stage III CRC with chemotherapy. (**A**) The overall survival and disease-free survival of patients less than 50 year-old. (**B**) The overall survival and disease-free survival of patients 50–75 year-old. (**C**) The overall survival and disease-free survival of patients over 75 year-old.

To investigate the association between age and chemotherapy irrespective of clinical tumor stage and to guide chemotherapy treatment for elderly patients with CRC, an analysis was performed with adjustment for AJCC stage II high-risk and stage III stratification. For the patients less than 50 year-old, there was no significant difference between age and chemotherapy (Fig. 3A). The outcomes of 50–75 year-old patients with FOLFOX or CapeOX were substantially better than those of patients without chemotherapy. The patients with stage II high-risk CRC treated with FOLFOX had better survival than those with other chemotherapies (Fig. 3B). FOLFOX and CapeOX achieved similar outcomes in patients with stage III CRC (Fig. 3B), and there was no significant difference between them. For the patients older than 75 years of age, likely, there was no evidence of a survival benefit for patients with stage III CRC with chemotherapy, probably because of deaths from other causes (Fig. 3C). However, it seemed implausible that patients with stage II high-risk CRC gained a survival benefit treated with FOLFLX or CapeOX, compared with those without chemotherapy (Fig. 3C).

All in all, our results regarding the survival benefit from chemotherapy for different stages and age of patients with CRC were in accordance with current NCCN guideline recommendations, except for no benefit from chemotherapy in less than 50 year-old patients.

### Pathological molecular markers and individualized chemotherapy

**Figure 4:**
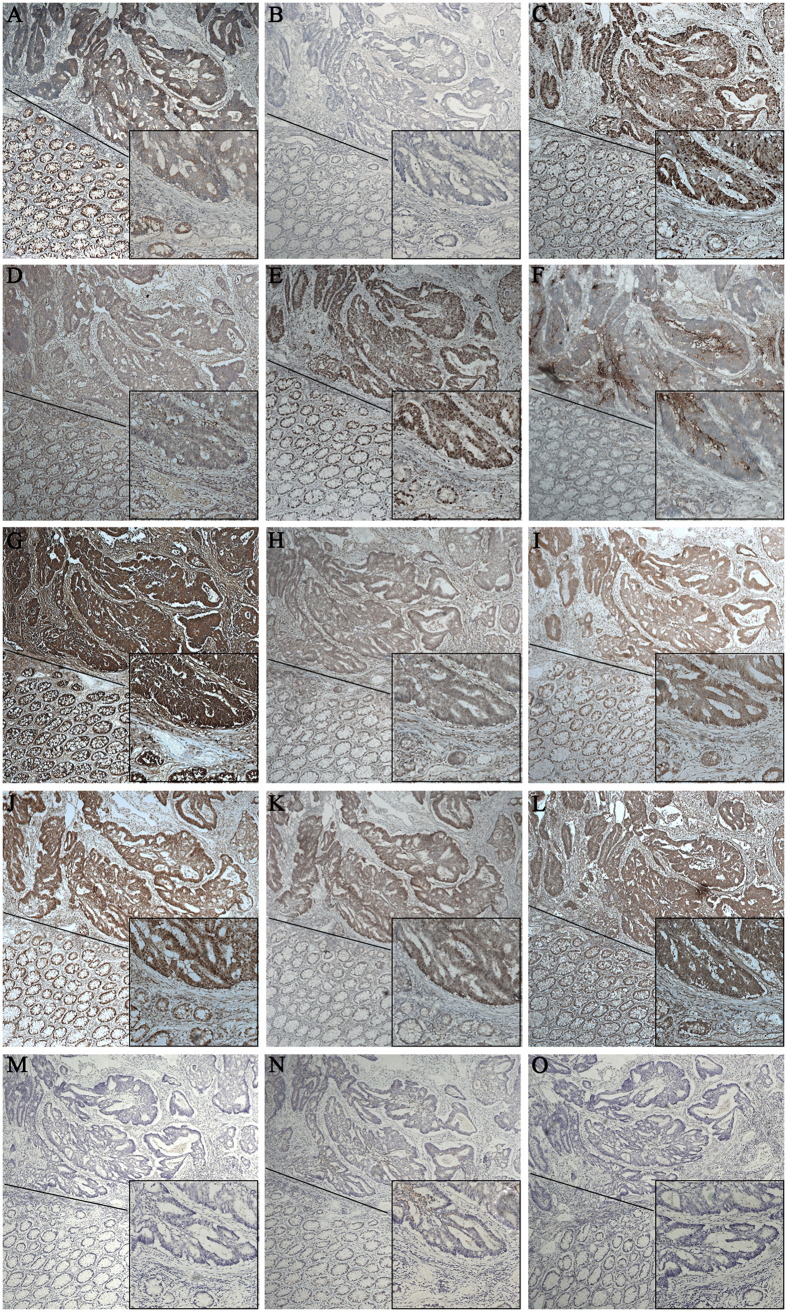
The immunohistochemical expression of 14 pathological markers at the same position of the same sample of CRC (both tumor tissues and adjacent normal mucosa in the same field). Original magnification ×50, ×200. (**A**) HER-2 expression. (**B**) EGFR expression. (**C**) TOPIIα expression. (**D**) P170 expression. (**E**) Ki67 expression. (**F**) CA199 expression. (**G**) CEA expression. (**H**) ERCC1 expression. (**I**) MLH1 expression. (**J**) MSH2 expression. (**K**) CDX2 expression. (**L**) Nm23 expression. (**M**) PTEN expression. (**N**) Survivin expression. (**O**) PBS (negative control).

Her-2, EGFR, TOPIIα, P170, Ki67, CA199, CEA, ERCC1, MLH1, MSH2, CDX2, nm23, PTEN and survivin are routine pathological molecular markers that are evaluated by immunohistochemistry in the department of pathology as required to diagnose CRC (Fig. 4). The detailed immunohistochemical expression of these 14 markers in our cohort is shown in Supplementary Table 5.

**Figure 5:**
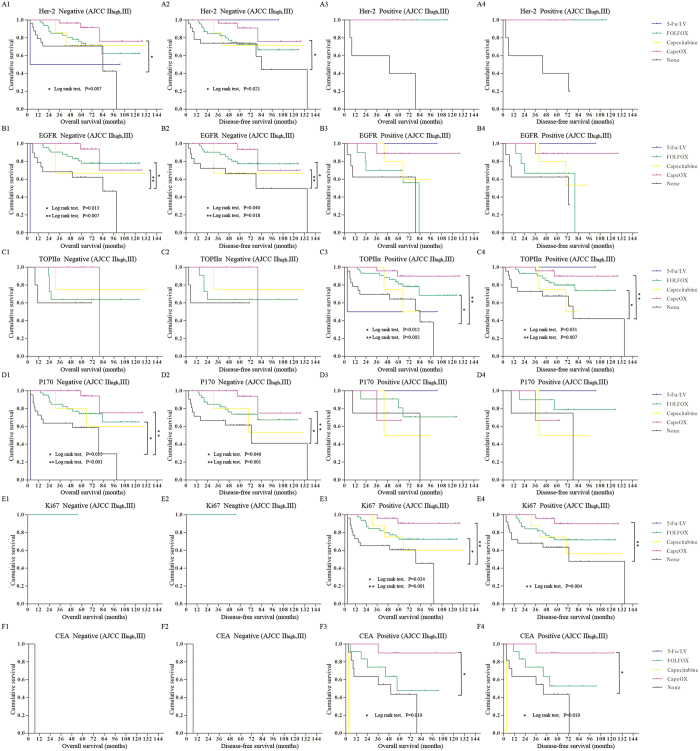
The Kaplan-Meier survival comparison of the patients with stage II high-risk and stage III CRC with chemotherapy grouped by expression of different pathological markers in our cohort. (**A**) The overall survival and disease-free survival based on Her-2 expression. (**B**) The overall survival and disease-free survival based on EGFR expression. (**C**) The overall survival and disease-free survival based on TOPIIα expression. (**D**) The overall survival and disease-free survival based on P170 expression. (**E**) The overall survival and disease-free survival based on Ki67 expression. (**F**) The overall survival and disease-free survival based on CEA expression.

To help guide individualized chemotherapy for stage II high risk and stage III CRC patients, we examined the impact that expression of these pathological markers had on patient survival. Her-2, EGFR, TOPIIα, P170, Ki67 and CEA were helpful for stratifying response to chemotherapy for patients with stage II high-risk and stage III CRC. CapeOX was the optimal adjuvant chemotherapy for individual patients when Her-2 was negative or Ki67, or CEA was positive (Fig. 5, Supplementary Fig. 2). Moreover, when EGFR or P170 was negative or TOPIIα was positive, FOLFOX or CapeOX gave the best outcomes (Fig. 5, Supplementary Fig. 2).

### TCGA samples characteristics

The colon adenocarcinoma databases from TCGA provided 458 cases, but 10 cases lacked complete clinical data. Therefore, 448 cases were examined in our study to validate the preliminary results from our cohort. The clinical characteristics of these 448 samples are shown in Fig. 1 and Table 1. Of the 448 samples, 35.1% were stage II high-risk and stage III cases. A total of 135 patients (30.1%) received adjuvant chemotherapy. The four chemotherapy regimens examined in the study were 5-Fu/LV (10 cases), FOLFOX (61 cases), capecitabine (12 cases) and CapeOX (2 cases).

We tested the TCGA samples’ representation using the Cox proportional hazards model. We found that age (P = 0.005; P = 0.004, respectively), pT stage (P = 0.018; P = 0.039, respectively), pN stage (P = 0.001) and distant metastasis (P = 0.001; P = 0.002, respectively) were significant independent prognostic factors for the OS and DFS, which is completely in accordance with the results from our cohort (Supplementary Table 6).

### Verification of individualized chemotherapy

To verify the accuracy of our results regarding individualized responses to chemotherapy, we divided the TCGA samples based on the expression of these 6 significant markers and evaluated the survival curves of each group. The detailed expression of these 6 significant markers in the TCGA was shown in Supplementary Table 7. However, there were only 28 stage II high-risk samples in the TCGA dataset, only 3 of which were from patients treated with chemotherapy, all with FOLFOX. Therefore, we excluded these 28 stage II high-risk samples to avoid statistical errors caused by sample deviation. Only 129 stage III samples were included to compare the survival rates of patients treated with four different chemotherapy regimens. Expression of EGFR, TOPIIα, P170, Ki67 and CEA were able to guide optimal chemotherapy choices. When expression of TOPIIα, Ki67, or CEA was positive or EGFR or P170 was negative, the patients with FOLFOX had better survival.

**Figure 6:**
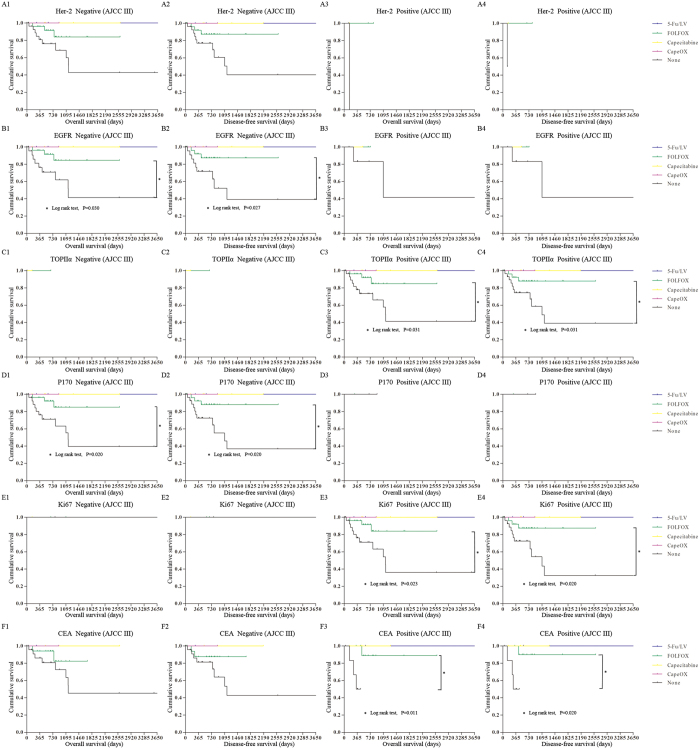
The Kaplan-Meier survival comparison of the stage III TCGA samples from patients treated with chemotherapy grouped by expression of 6 significantly pathological markers expression. (**A**) The overall survival and disease-free survival based on Her-2 expression. (**B**) The overall survival and disease-free survival based on EGFR expression. (**C**) The overall survival and disease-free survival based on TOPIIα expression. (**D**) The overall survival and disease-free survival based on P170 expression. (**E**) The overall survival and disease-free survival based on Ki67 expression. (**F**) The overall survival and disease-free survival based on CEA expression.

We confirmed that expression of TOPIIα, EGFR and P170 could be potential predictive markers of individualized chemotherapy response, combined with our preliminary conclusions in the end. When TOPIIα was positive or EGFR or P170 was negative, FOLFOX was the optimal adjuvant chemotherapy for patients with stage II high-risk and stage III CRC (Fig. 6).

**Figure 7:**
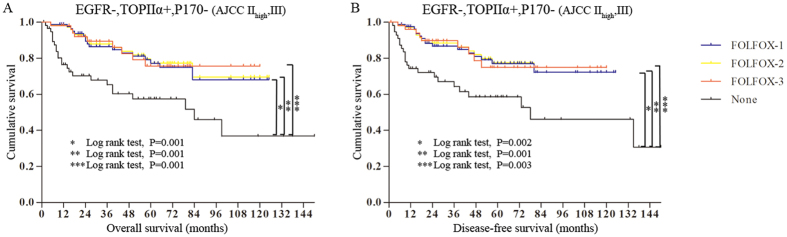
The Kaplan-Meier survival comparison of patients of our cohort and TCGA database together treated with FOLFLX grouped by combined markers of EGFR-, TOPIIα+ and P170-. FOLFLX1: at least one of three expressions (EGFR-, TOPIIα+ and P170-); FOLFLX 2: at least two of three expressions (EGFR-, TOPIIα+ and P170-); FOLFLX3: simultaneous expressions of EGFR-, TOPIIα+ and P170-. (**A**) The overall survival of combined markers of EGFR-, TOPIIα+ and P170-. (**B**) The disease-free survival of combined markers of EGFR-, TOPIIα+ and P170-.

Furthermore, we combined our cohort with TCGA database to increase the amount of the samples to verify the accuracy of guiding FOLFOX by these three markers (EGFR-, TOPIIα+ and P170-). The combined samples were divided into three groups in regard to their expressions: group 1, at least one of three expressions (EGFR-, TOPIIα+ and P170-); group 2, at least two of three expressions (EGFR-, TOPIIα+ and P170-); and group 3, simultaneous expressions of EGFR-, TOPIIα+ and P170-. It was showed those patients with simultaneous expressions of EGFR-, TOPIIα+ and P170- with FOLFLX had better outcomes than patients only with one or two of them expressions (Fig. 7).

Therefore, combined these three markers (EGFR-, TOPIIα+ and P170-) would have more power to guide individualized chemotherapy with FOLFLX than any single one alone.

## Discussion

Adjuvant chemotherapy is effective for patients with resected, stage II and III CRC^6^. According to the latest NCCN recommendations, FOLFOX or CapeOX is recommended as a preferred treatment for stage III CRC. However, in stage II CRC, the benefit of adding of oxaliplatin to 5-Fu/LV or capecitabine in adjuvant therapy has not been demonstrated. Moreover, for patients 70 years of age and older, treatment with FOLFOX has not proven to provide a survival benefit compared with 5-FV/LV^10^. Although the guidelines provide a clear direction for chemotherapy for patients with stage II high-risk and stage III CRC, they are insufficient for individualizing patient treatment. For example, it is still controversial whether or not to offer adjuvant chemotherapy to patients 70 years of age and older. Likewise, it is still unclear whether FOLFOX or CapeOX would provide better survival for individual patients.

The purpose of our study was to investigate whether survival outcomes for Chinese patients of different stages and ages treated with chemotherapy were consistent with the guidelines and to explore how to guide individualized chemotherapy using pathological markers.

We discovered that the patient composition and proportion receiving chemotherapy in our cohort was in accordance with that of other large Chinese samples based on Cox proportional hazards analysis. Therefore, the results from our cohort were representative of the larger population. Based on our results, FOLFOX or CapeOX was recommended as a preferred treatment for patients with stage II high-risk and stage III but not for those with stage II low-risk disease. Treatment with FOLFOX or CapeOX may provide significantly better long-term survival benefit than those without chemotherapy, which was in accordance with the guidelines. In evaluating age, we found that patients older than 75 years of age with stage II high-risk CRC might experience a survival benefit from chemotherapy.

In fact, CRC is a disease of the elderly. More than one-third of all deaths (29% in men and 43% in women) will occur in individuals 80 years of age and older^2^. Whether or not to use chemotherapy for elderly patients presents a common and substantial challenge. Two retrospective analyses from Surveillance, Epidemiology, and End Results (SEER)-Medicare Database found a survival benefit with chemotherapy for elderly patients^25^,^26^,^27^. However, the benefit for elderly patients from chemotherapy came at the cost of increased toxicity, particularly paresthesias, bone marrow toxicity, and gastrointestinal toxicity^12^,^28^. Therefore, the decision whether or not to use chemotherapy for elderly patients should be comprehensively based on individual status and preference, not just tumor stage. Indeed, there is an urgent need to individualize chemotherapy for elderly patients. Our data demonstrated that only elderly patients with stage II high-risk not stage III CRC might had a survival benefit with chemotherapy, weighing the benefits and side effects of chemotherapy.

The concept of individualized chemotherapy has gained widespread acceptance^29^. CRC is a heterogeneous disease with widely varied outcomes. Even patients with similar clinical and pathological features receiving the same chemotherapy may have different outcomes. Individualized chemotherapy for CRC is a major trend, especially in the era of precision medicine. Every decision regarding chemotherapy should be based upon the individual patient and his tumor characteristics. However, there is no simple method to process how to individualize chemotherapy for every patient. Detection of KRAS, NRAS or BRAF mutations for individual CRC patients has been proven predictive for whether or not to use cetuximab or panitumumab in combination with standard chemotherapy^10^,^14^. However, the NCCTG intergroup phase III trial N0147 and PETACC-8 trial found no additional benefit to add these targeted agents to standard chemotherapy^15^,^16^,^17^,^18^. Several multigene assays have been developed in hopes of providing prognostic and predictive information to aid in individualizing chemotherapy for patients with stage II high-risk and stage III CRC, especially for elderly patients^30^,^31^,^32^,^33^,^34^,^35^. Unfortunately, these multigene assays, Oncotype DX, ColoPrint and ColDx, are only prognostic for recurrence, DFS and OS but are not predictive for benefit from chemotherapy^12^,^36^,^37^. Validated predictive markers for benefit from chemotherapy benefit for patients with stage II high-risk and stage III CRC are lacking^29^. Furthermore, individualized chemotherapy for CRC requires a deep understanding of tumor biology and a clear identification for subsets of tumors.

Pathological molecules such as Her-2, EGFR, P170, Ki67, etc., are routinely tested in the department of pathology of every hospital as required to diagnose CRC. Moreover, these pathological markers may not only improve more accurate diagnosis subgroups of CRC and define tumor characteristics, but also guide clinical therapy for CRC in a convenient, fast and inexpensive way. Therefore, pathological markers may help us to select optimal chemotherapy with maximizing the benefit of treatment and minimizing toxicity, serving as potential predictive markers for individualized chemotherapy.

In fact, there is a broad consensus that breast cancer therapy has become more and more individualized on the basis of pathological molecular markers such as estrogen receptor (ER), progesterone receptor (PR) and HER-2^38^. By detecting the status of ER, PR and HER-2, doctors can make decision on whether and what type of endocrine therapy or chemotherapy might be most effective^39^. Therefore, it is possible to individualize chemotherapy for patients with stage II high-risk and stage III CRC by detecting expression of these pathological markers. Our study revealed that Her-2, EGFR, TOPIIα, P170, Ki67 and CEA seemed to be helpful to guide chemotherapy. CapeOX was the optimal adjuvant chemotherapy for individual patients when Her-2 was negative or Ki67, or CEA was positive. When EGFR or P170 was negative or TOPIIα was positive, FOLFOX or CapeOX was recommended as a preferred treatment. However, these results were obtained only by our hospital’s cohort and needed to be validated and possibly revised.

TCGA is a publicly available dataset, that can help to improve diagnostic methods, treatment standards, and finally to prevent cancers^40^,^41^. Every researcher can freely analyze gene expression data with clinical data from TCGA to predict prognosis and find biomarkers for all kinds of cancers^42^. We downloaded data of 458 colon adenocarcinoma samples from TCGA to verify our preliminary conclusions. For these 6 significant pathological markers, we evaluated their expression by replacing protein level measured by immunohistochemistry with mRNA level measured by Hiseq in the TCGA samples. Finally, we confirmed that TOPIIα, EGFR and P170 could be potential predictive markers for individualized chemotherapy. FOLFOX was the optimal adjuvant chemotherapy for patients with stage II high-risk and stage III CRC when TOPIIα was positive or EGFR or P170 was negative. Furthermore, combined these three markers (EGFR-, TOPIIα+ and P170-) would have more power to guide individualized chemotherapy with FOLFLX than any single one alone.

The significance of our final results will be in helping to guide individualized chemotherapy in a convenient, fast and inexpensive way in routine clinical practice for those confusing patients for whom FOLFOX or CapeOX seem to have equivalent efficacy or for elderly patients with a strong preference for chemotherapy.

Indeed, we cannot make decision on chemotherapy for CRC completely on the basis of pathological markers, as is done in breast cancer treatment. However, it is absolutely possible to help discover more sensitive and effective chemotherapy for individuals and to define the optimal adjuvant chemotherapy for patients in routine clinical practice by detecting TOPIIα, EGFR and P170 at the same time of pathologic diagnosis in a convenient, fast and inexpensive way.

In summary, our results regarding the survival benefit for chemotherapy in patients of different stages and ages were consistent with the latest guidelines. In addition, our data added to the evidence that chemotherapy might be used for elderly patients with stage II high-risk CRC. Furthermore, this is the first report to guide individualized chemotherapy for patients with CRC by different pathological markers at the same time of pathologic diagnosis. FOLFOX is the optimal adjuvant chemotherapy for patients with stage II high-risk and stage III CRC when TOPIIα is positive or EGFR or P170 is negative.

Our study is based on a retrospective analysis of the data from our hospital and TCGA. The retrospective nature and relatively small sample size have limited power to predict prospective outcomes. Therefore, these results still need to be verified in several multi-center randomized, controlled trials.

## Methods

### Ethics, consent and permissions

The clinical protocol of this study was reviewed and approved by the Institutional Review Boards of Shanghai General Hospital Affiliated to Shanghai Jiao Tong University (No. 2014KY046). Informed consent was obtained from all subjects before their participation in the study, and the methods were strictly carried out in accordance with the approved guidelines.

### Patients data

The data of 953 patients who underwent curative surgery for CRC at Shanghai General Hospital from January 2003 through December 2010 were collected in our study. However, excluding those with incomplete information for clinicopathologic features or who were treated with preoperative chemotherapy, a total of 902 patients (448 men and 454 women) were included in the study. The distribution of staging for these cases was American Joint Committee on Cancer (AJCC) stage I, 162 patients; II, 362 patients; III, 289 patients and IV, 89 patients. All diagnoses were confirmed by two pathologists, and the tumor grade and stage classifications were assigned according to the AJCC system^43^. The clinical and pathologic features studied for patients with CRC included age, sex, tumor location, primary tumor size, tumor differentiation, TMN stage, primary tumor classification, and adjuvant chemotherapy given. Regular patient follow-up was achieved by annual telephone interviews by the surgical team, last conducted in December 2014. Follow-up evaluation included the following end-points: overall survival (OS, any death), and disease free survival (DFS, first recurrence/relapse after surgical treatment).

### Pathological markers data

The 14 pathological markers were Her-2, EGFR, TOPIIα, P170, Ki67, CA199, CEA, ERCC1, MLH1, MSH2, CDX2, nm23, PTEN and survivin. Their immunohistochemical expressions in the tumor samples from 300 patients with CRC were provided by the department of pathology of our hospital. The evaluation was based on staining intensity and extension as described previously^44^.

### TCGA colon adenocarcinoma database

All of the 458 validation samples are based on data generated by The Cancer Genome Atlas (TCGA) Research Network (http://cancergenome.nih.gov/). These data include mRNA expression and clinical characteristics. For each of 6 pathologic markers (Her-2, EGFR, TOPIIα, P170, Ki67 and CEA), the cancer population could be divided into positive and negative groups based on the gene expression being greater or less than that of the control group. The survival of two patient subgroups for each gene was compared and tested with the log-rank test.

### Statistical analysis

All data and survival analyses were performed using SPSS 13.0 statistical software (SPSS, Chicago, USA). Patient and tumor characteristics were summarized with means ± standard deviation for continuous variables, and with frequencies and percentage for categorical variables. The survival rates were calculated by the Kaplan-Meier method and the differences between the survival curves were examined by the log-rank test. The Cox proportional hazards model was used for multivariate analysis and to investigate independent prognostic factors. All P-values < 0.05 were considered statistically significant.

## Additional Information

**How to cite this article**: Han, Y. *et al.* A comparative analysis and guidance for individualized chemotherapy of stage II and III colorectal cancer patients based on pathological markers. *Sci. Rep.* **6**, 37240; doi: 10.1038/srep37240 (2016).

**Publisher’s note:** Springer Nature remains neutral with regard to jurisdictional claims in published maps and institutional affiliations.

## Supplementary Material

Supplementary Information

## Figures and Tables

**Table 1:** Clinicopathologic Features of Patients With CRC in our cohort and TCGA samples.

	Our hospital cohort	TCGA cohort
	N = 902	N = 448
Age		
<50 years	79 (8.8%)	52 (11.6%)
50–75 years	554 (61.4%)	250 (55.8%)
>76 years	269 (29.8%)	146 (32.6%)
Sex		
Male	448 (49.7%)	238 (53.1%)
Female	454 (50.3%)	210 (46.9%)
Tumor size		
<4 cm	308 (34.1%)	—
4–6 cm	436 (48.3%)	—
>6 cm	158 (17.5%)	—
Tumor location		
Ascending	214 (23.7%)	197 (44.0%)
Transverse	62 (6.9%)	78 (17.4%)
Descending	59 (6.5%)	20 (4.5%)
Sigmoid	219 (24.3%)	153 (34.2%)
Rectum	348 (38.6%)	—
T stage		
T1	49 (5.4%)	10 (2.2%)
T2	157 (17.4%)	78 (17.4%)
T3	454 (50.3%)	307 (68.5%)
T4	242 (26.8%)	53 (11.8%)
N stage		
N0	554 (61.4%)	266 (59.4%)
N1	245 (27.2%)	101 (22.5%)
N2	103 (11.4%)	81 (18.1%)
M stage		
M0	813 (90.1%)	385 (85.9%)
M1	89 (9.9%)	63 (14.1%)
AJCC stage		
I	162 (18%)	77 (17.2%)
II_low_	263 (29.1%)	151 (33.7%)
II_high_*	99 (11.0%)	28 (6.3%)
III	289 (32.0%)	129 (28.8%)
IV	89 (9.9%)	63 (14.1%)
Differentiation		
Well	336 (37.3%)	—
Moderate	519 (57.5%)	—
Poorly	47 (5.2%)	—
Vascular invasion		
No	810 (89.8%)	—
Yes	92 (10.2%)	—
Chemotherapy		
No	299 (33.1%)	313 (69.9%)
Yes	603 (66.9%)	135 (30.1%)
5-Fu/LV	37 (6.1%)	10 (7.4%)
FLOFLX	392 (65%)	61 (45.2%)
Capecitabine	50 (8.3%)	12 (8.9%)
CapeOX	102 (16.9%)	2 (1.5%)
others	22 (3.6%)	50 (37%)

^*^Patients with high-risk stage II disease, defined as those with poor prognostic features, including T4 tumors; inadequately sampled node (<12 lymph nodes) *et al.*
