# Supplementary material for: A comparative analysis and guidance for individualized chemotherapy of stage II and III colorectal cancer patients based on pathological markers
Source: Sci Rep. 2016 Nov 15;6:37240. doi: 10.1038/srep37240 (PMC5109035; doi:10.1038/srep37240)
Supplement: Supplementary Information [file srep37240-s1.doc]

**A comparative analysis and guidance for individualized chemotherapy of stage II and III colorectal cancer patients based on pathological markers**

Yang Han^1,+^, Su Lu^2,+^, Fudong Yu^1^, Xisheng Liu^1^, Huiming Sun^2^, Jingtao Wang^1^, Xingwu Zhu^1^, Huijun Lu^2^, Hao Yue^2^, Jing Wang^2^, Jun Lin^2^, Chongzhi Zhou^1^, Huamei Tang^2,*^ & Zhihai Peng^1,*^

1 Department of General Surgery, Shanghai General Hospital, School of Medicine, Shanghai Jiao Tong University, Shanghai 20080

2 Department of Pathology, Shanghai General Hospital, School of Medicine, Shanghai Jiao Tong University, Shanghai 20080

+ Yang Han and Su Lu are regarded as co-first author.

*Corresponding author: Hua-mei Tang

Email: [tanghuamei2014@163.com](mailto:tanghuamei2014@163.com)

Address: Department of Pathology, Shanghai General Hospital, School of Medicine, Shanghai Jiao Tong University, 100 Haining Road, Shanghai 20080

Tel and Fax: +86-21-36126213

*Corresponding author: Zhi-hai Peng

Email: [pengzhihai0801@163.com](mailto:pengzhihai0801@163.com)

Address: Department of General Surgery, Shanghai General Hospital, School of

Medicine, Shanghai Jiao Tong University, 100 Haining Road, Shanghai 20080

Tel and Fax: +86-21-36126213


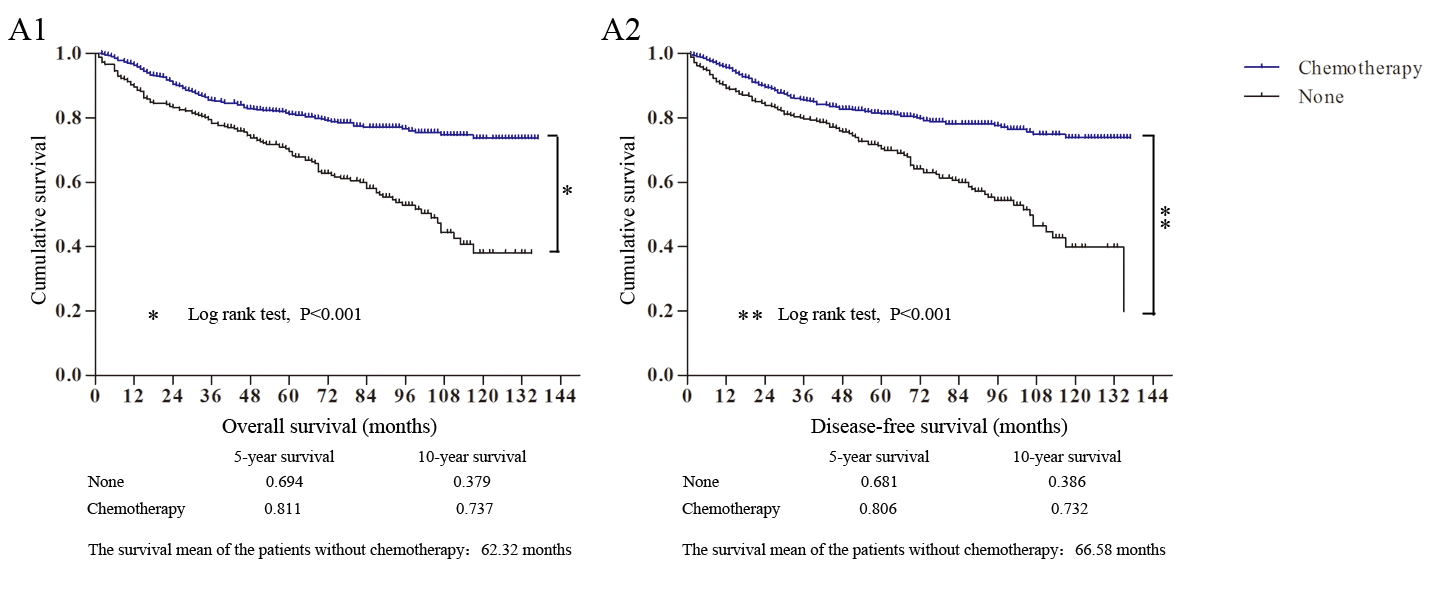


Supplementary Figure.1 The Kaplan-Meier survival comparison of the patients with and without chemotherapy in our cohort.

A: The overall survival of the patients with and without chemotherapy.

B: The disease-free survival of the patients with and without chemotherapy.


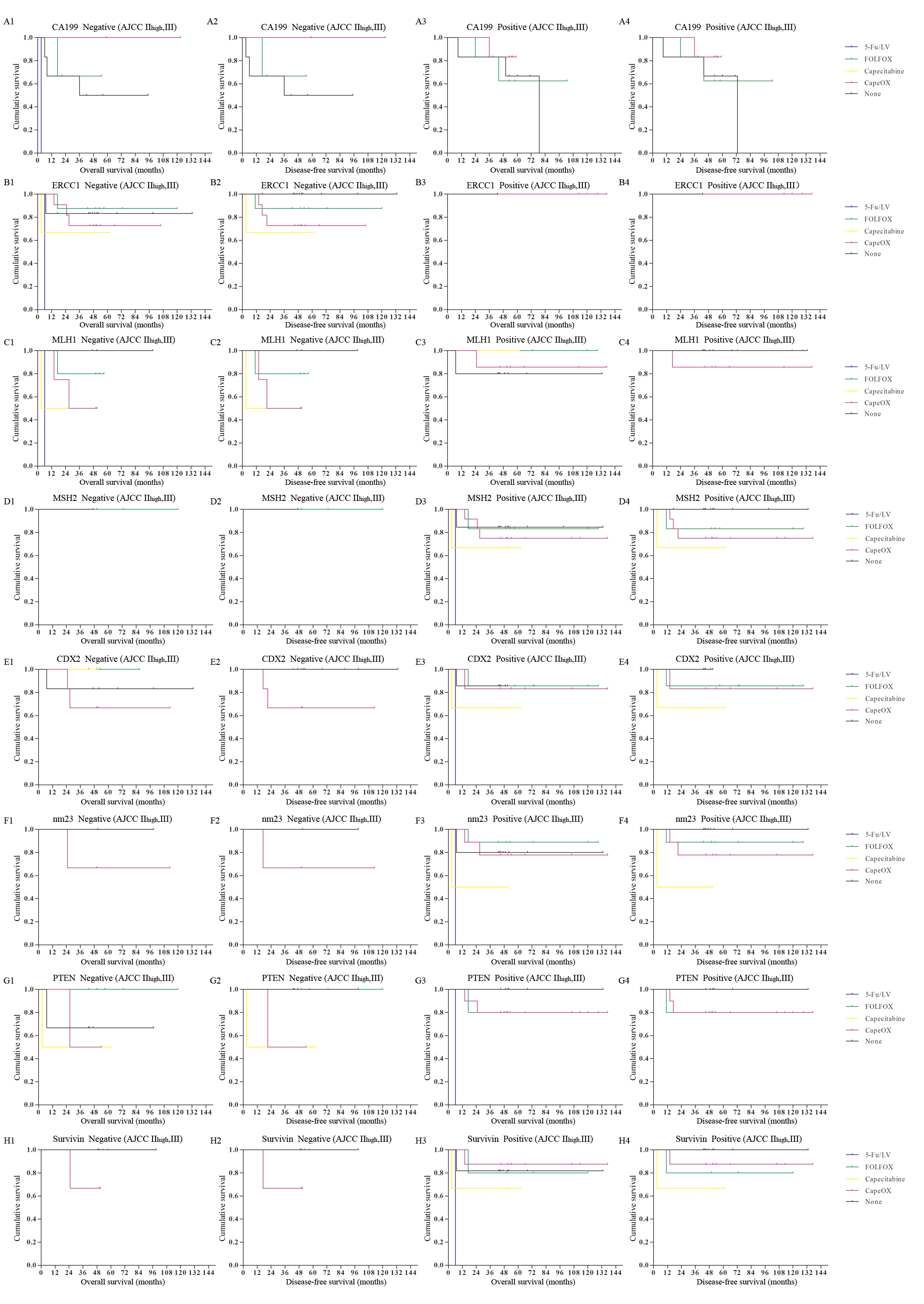


Supplementary Figure.2 The Kaplan-Meier survival comparison of the patients with stage II high-risk and stage III CRC with chemotherapy grouped by expression of different pathological markers in our cohort.

A: The overall survival and disease-free survival based on CA199 expression.

B: The overall survival and disease-free survival based on ERCC1 expression. C: The overall survival and disease-free survival based on MLH1 expression.

D: The overall survival and disease-free survival based on MSH2 expression.

E: The overall survival and disease-free survival based on CDX2 expression.

F: The overall survival and disease-free survival based on nm23 expression.

G: The overall survival and disease-free survival based on PTEN expression.

H: The overall survival and disease-free survival based on Survivin expression.


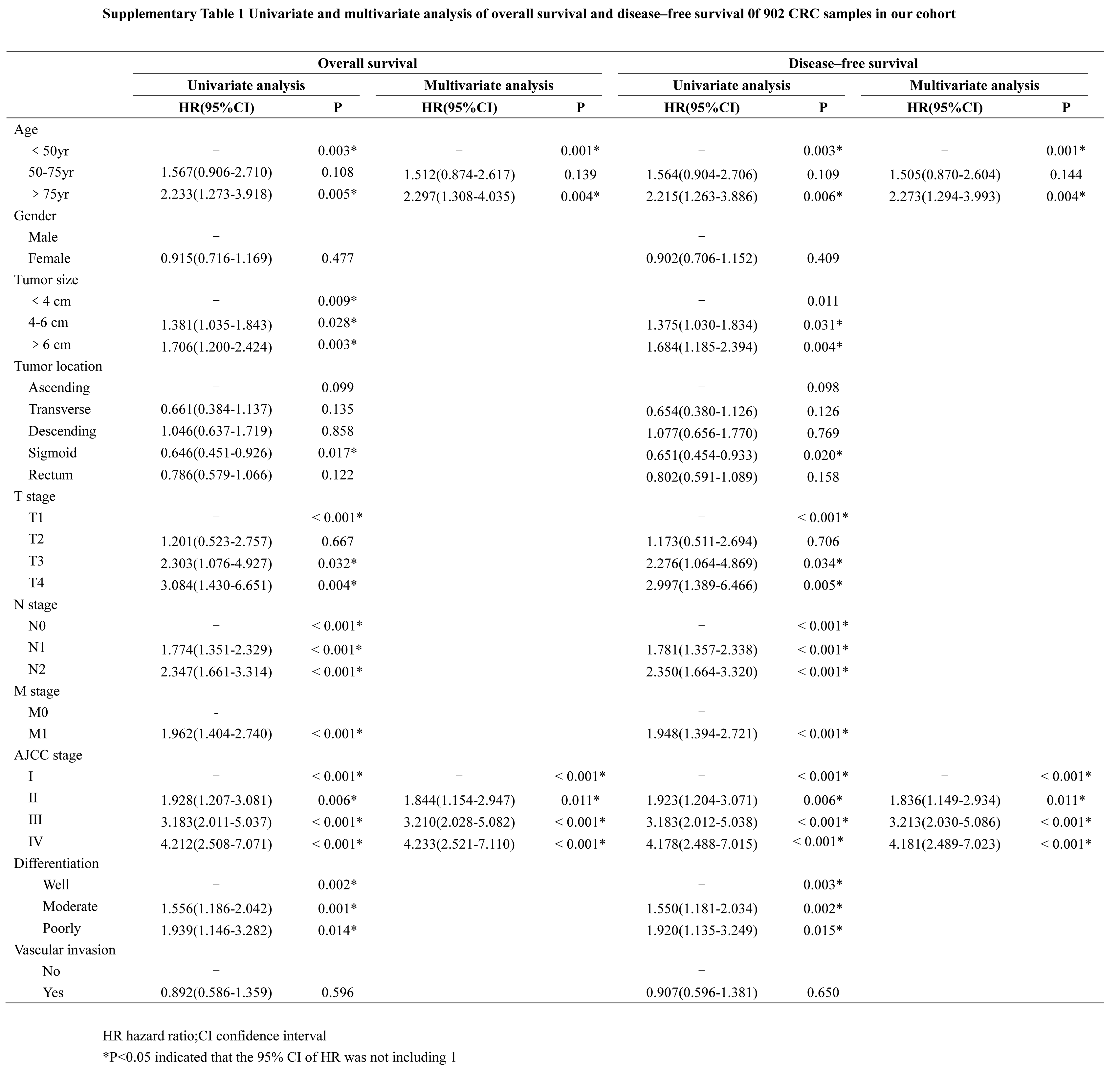


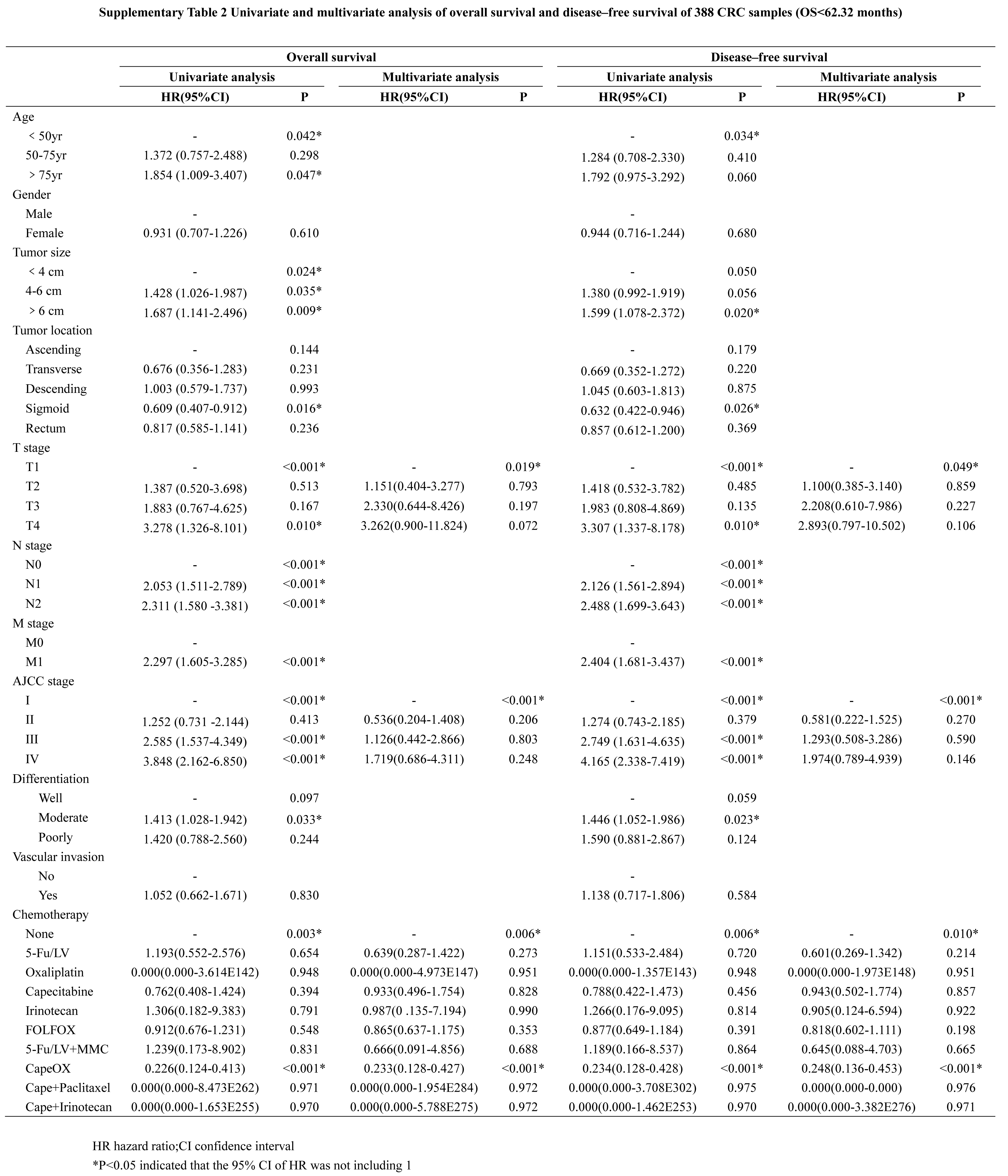


Supplementary Table 3. Adjuvant chemotherapy for CRC patients in our cohort (N=902)

|  | Adjuvant Chemotherapy | N |
| --- | --- | --- |
| No | None | 299 |
| Single-agent | 5-Fu/LV | 37 |
|  | Oxaliplatin | 7 |
|  | Capecitabine | 50 |
|  | Irinotecan hydrochloride | 1 |
|  | FT207 | 2 |
| Combination therapies | 5-Fu/LV+ Oxaliplatin | 392 |
|  | 5-Fu/LV+MMC | 6 |
|  | 5-Fu/LV+Irinotecan hydrochloride | 2 |
|  | Capecitabine+ Oxaliplatin | 102 |
|  | Capecitabine+ Paclitaxel | 1 |
|  | Capecitabine+5-Fu/LV | 2 |
|  | Capecitabine+ Irinotecan hydrochloride | 1 |


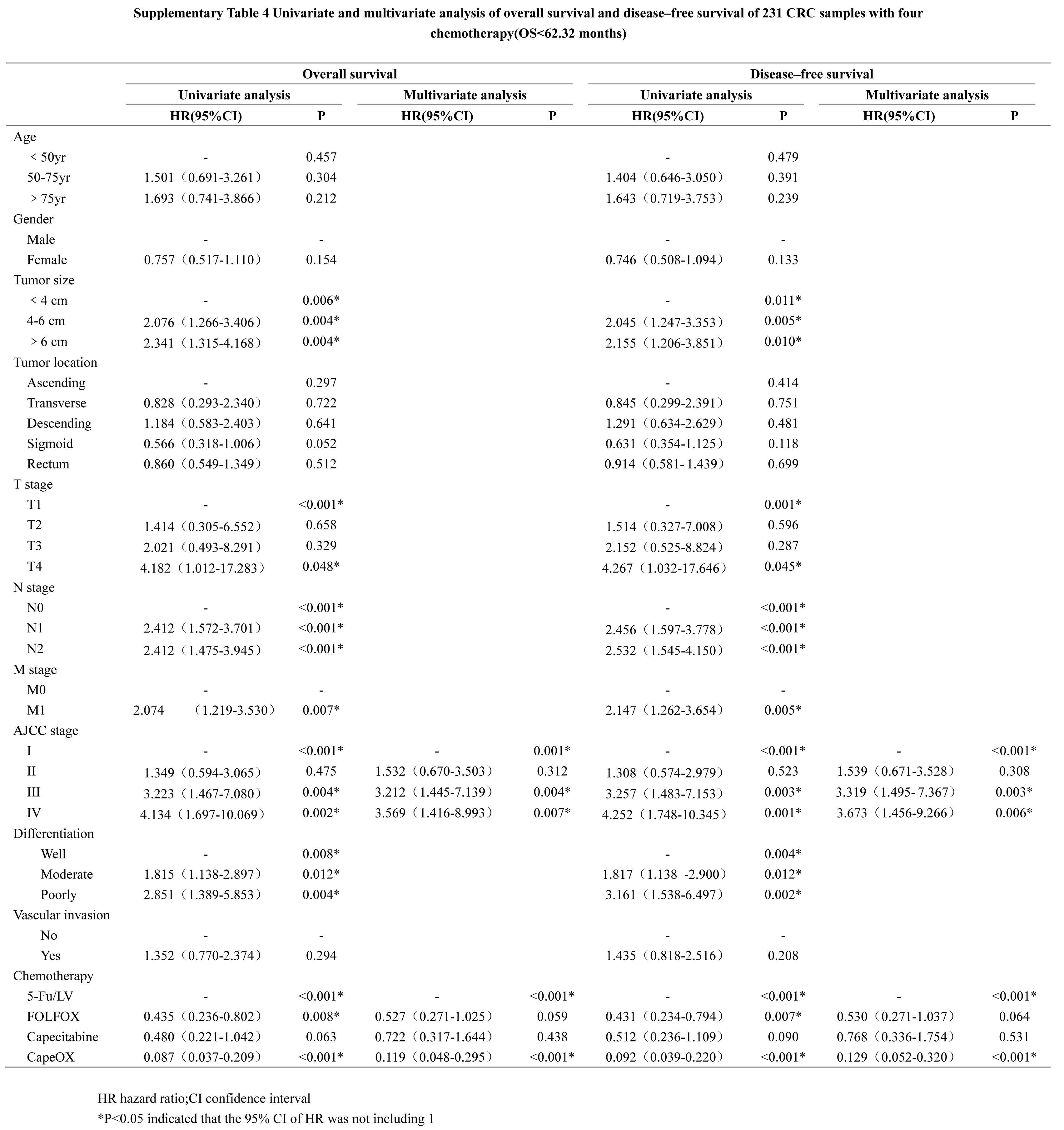


Supplementary Table 5 The expression of 15 pathological markers in CRC patients with four kinds of adjuvant chemotherapy in our cohort.

|  |  | None | 5-Fu/LV | FOLFOX | Capecitabine | CapeOX |
| --- | --- | --- | --- | --- | --- | --- |
| Marker | N | (N=299) | (N=37) | (N=392) | (N=50) | (N=102) |
| Her-2 | 189 |  |  |  |  |  |
| Negative |  | 37(19.6%) | 4(2.1%) | 69(36.5%) | 18(9.5%) | 41(21.7%) |
| Positive |  | 5(2.6%) | 0(0%) | 10(5.3%) | 2(1.1%) | 3(1.6%) |
| EGFR | 191 |  |  |  |  |  |
| Negative |  | 29(15.2%) | 3(1.6%) | 63(33.0%) | 10(5.2%) | 32(16.8%) |
| Positive |  | 11(5.8%) | 2(1.0%) | 18(9.4%) | 11(5.8%) | 12(6.3%) |
| TOPIIα | 193 |  |  |  |  |  |
| Negative |  | 11(5.7%) | 1(0.5%) | 18(9.3%) | 4(2.1%) | 10(5.2%) |
| Positive |  | 30(15.5%) | 4(2.1%) | 65(33.7%) | 17(8.8%) | 33(17.1%) |
| P170 | 185 |  |  |  |  |  |
| Negative |  | 33(17.8%) | 4(2.2%) | 67(36.2%) | 16(8.6%) | 36(19.5%) |
| Positive |  | 6(3.2%) | 1(0.5%) | 12(6.5%) | 4(2.2%) | 6(3.2%) |
| P53 | 189 |  |  |  |  |  |
| Negative |  | 11(5.8%) | 1(0.5%) | 28(14.8%) | 9(4.8%) | 18(9.5%) |
| Positive |  | 30(15.9%) | 4(2.1%) | 52(27.5%) | 11(5.8%) | 25(13.2%) |
| Ki67 | 170 |  |  |  |  |  |
| Negative |  | 0(0%) | 0(0%) | 2(1.2%) | 0(0%) | 1(0.6%) |
| Positive |  | 38(22.4%) | 4(2.4%) | 69(40.6%) | 20(11.8%) | 36(21.2%) |
| CA199 | 56 |  |  |  |  |  |
| Negative |  | 9(16.1%) | 1(1.8%) | 8(14.3%) | 2(3.6%) | 5(8.9%) |
| Positive |  | 9(16.1%) | 1(1.8%) | 8(14.3%) | 3(5.4%) | 10(17.9%) |
| CEA | 64 |  |  |  |  |  |
| Negative |  | 1(1.6%) | 0(0%) | 0(0%) | 0(0%) | 0(0%) |
| Positive |  | 18(28.1%) | 2(3.1%) | 20(31.3%) | 6(9.4%) | 17(26.6%) |
| ERCC1 | 79 |  |  |  |  |  |
| Negative |  | 22(27.8%) | 1(1.3%) | 15(19.0%) | 4(5.1%) | 20(25.3%) |
| Positive |  | 8(10.1%) | 0(0%) | 3(3.8%) | 2(2.5%) | 4(5.1%) |
| MLH1 | 76 |  |  |  |  |  |
| Negative |  | 8(10.5%) | 1(1.3%) | 9(11.8%) | 3(3.9%) | 9(11.8%) |
| Positive |  | 21(27.6%) | 0(0%) | 9(11.8%) | 3(3.9%) | 13(17.1%) |
| MSH2 | 79 |  |  |  |  |  |
| Negative |  | 4(5.1%) | 0(0%) | 7(8.9%) | 1(1.3%) | 1(1.3%) |
| Positive |  | 26(32.9%) | 1(1.3%) | 11(13.9%) | 5(6.3%) | 23(29.1%) |
| CDX2 | 80 |  |  |  |  |  |
| Negative |  | 11(13.7%) | 0(0%) | 10(12.5%) | 2(2.5%) | 8(10%) |
| Positive |  | 18(22.5%) | 1(1.3%) | 10(12.5%) | 4(5%) | 16(20%) |
| nm23 | 74 |  |  |  |  |  |
| Negative |  | 2(2.7%) | 0(0%) | 2(2.7%) | 2(2.7%) | 4(5.4%) |
| Positive |  | 26(35.1%) | 1(1.4%) | 14(18.9%) | 4(5.4%) | 19(25.7%) |
| PTEN | 77 |  |  |  |  |  |
| Negative |  | 10(13.0%) | 0(0%) | 7(9.1%) | 2(2.6%) | 6(7.8%) |
| Positive |  | 18(23.4%) | 1(1.3%) | 11(14.3%) | 4(5.2%) | 18(23.4%) |
| Survivin | 72 |  |  |  |  |  |
| Negative |  | 4(5.6%) | 0(0%) | 6(8.3%) | 1(1.4%) | 6(8.3%) |
| Positive |  | 23(31.9%) | 1(1.4%) | 10(13.9%) | 5(6.9%) | 16(22.2%) |


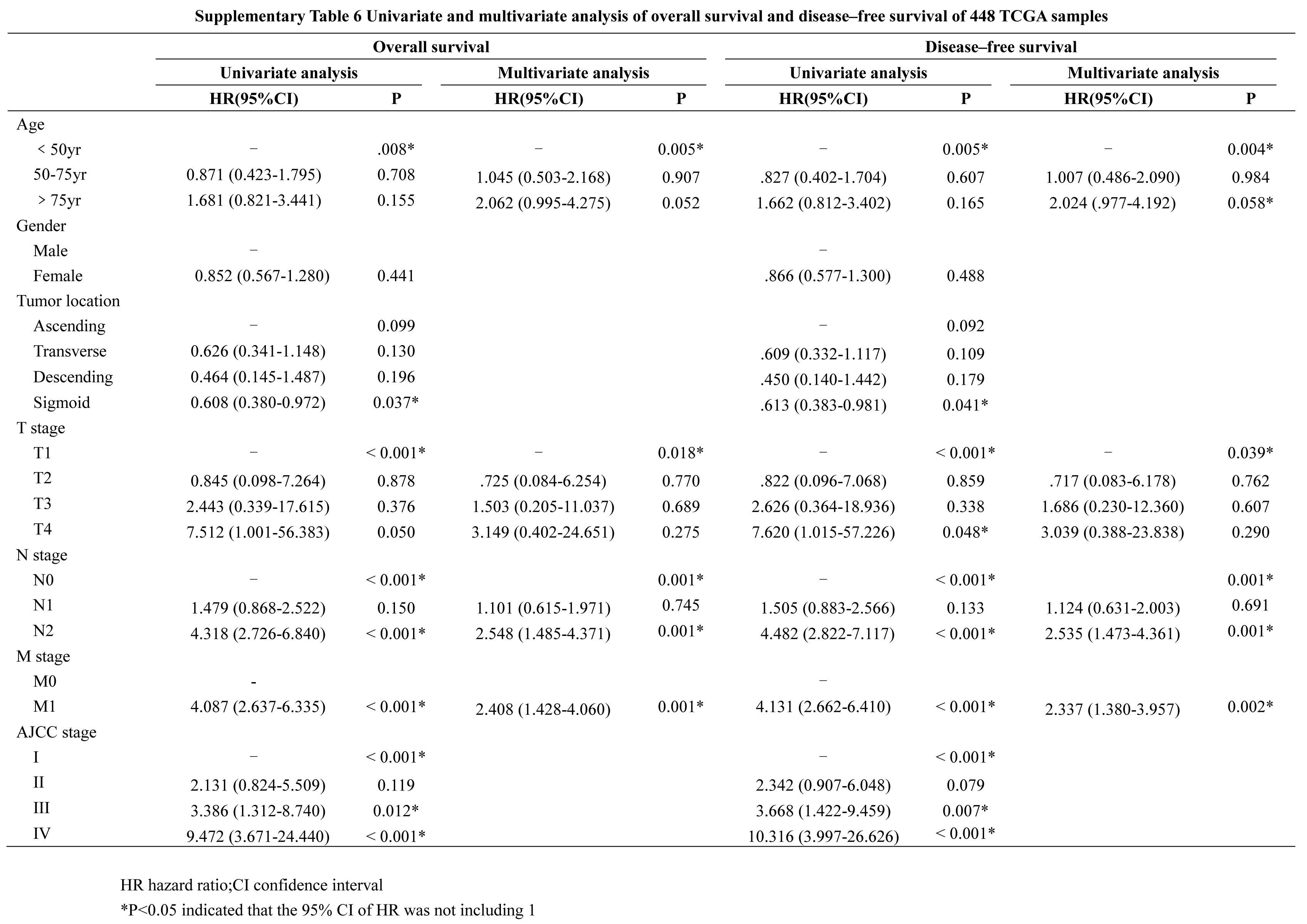


Supplementary Table 7 The expression of 6 pathological markers in the TCGA samples

|  | N | None | 5-Fu/LV | FOLFOX | Capecitabine | CapeOX |
| --- | --- | --- | --- | --- | --- | --- |
| Marker | 233 | 178 | 6 | 39 | 9 | 1 |
| Her-2 |  |  |  |  |  |  |
| Negative | 220 | 170 (77.3%) | 6 (2.7%) | 36 (16.4%) | 7 (3.2%) | 1 (0.5%) |
| Positive | 13 | 8 (61.5%) | 0 (0.0%) | 3(23.1%) | 2 (15.4%) | 0 (0.0%) |
| EGFR |  |  |  |  |  |  |
| Negative | 210 | 159 (75.7%) | 5 (2.4%) | 37 (17.6%) | 8 (3.8%) | 1(0.5%) |
| Positive | 23 | 19 (82.6%) | 1 (4.3%) | 2 (8.7%) | 1 (4.3%) | 0(0.0%) |
| TOPIIα |  |  |  |  |  |  |
| Negative | 6 | 4 (66.7%) | 0 (0.0%) | 1 (16.7%) | 1 (16.7%) | 0(0.0%) |
| Positive | 227 | 174 (76.7%) | 6 (2.6%) | 38 (16.7%) | 8 (3.5%) | 1(0.4%) |
| P170 |  |  |  |  |  |  |
| Negative | 218 | 165 (75.7%) | 6 (2.8%) | 37 (17.0%) | 9 (4.1%) | 1 (0.5%) |
| Positive | 15 | 13 (86.7%) | 0 (0.0%) | 2 (13.3%) | 0 (0.0%) | 0 (6) |
| Ki67 |  |  |  |  |  |  |
| Negative | 21 | 17 (81.0%) | 0 (0.0%) | 3 (14.3%) | 1 (4.8%) | 0 (0.0%) |
| Positive | 212 | 161 (75.9%) | 6 (2.8%) | 36 (17.0%) | 8 (3.8%) | 1 (0.5%) |
| CEA |  |  |  |  |  |  |
| Negative | 156 | 124 (79.5%) | 3 (1.9%) | 22 (14.1%) | 6 (3.8%) | 1 (0.6%) |
| Positive | 77 | 54 (70.1%) | 3 (3.9%) | 17 (22.1%) | 3 (3.9%) | 0 (23) |
